# Germline mismatch repair gene variants analyzed by universal sequencing in Japanese cancer patients

**DOI:** 10.1002/cam4.2432

**Published:** 2019-08-06

**Authors:** Yoshimi Kiyozumi, Hiroyuki Matsubayashi, Yasue Horiuchi, Satomi Higashigawa, Takuma Oishi, Masato Abe, Sumiko Ohnami, Kenichi Urakami, Takeshi Nagashima, Masatoshi Kusuhara, Hidehiko Miyake, Ken Yamaguchi

**Affiliations:** ^1^ Division of Genetic Medicine Promotion Shizuoka Cancer Center Shizuoka Japan; ^2^ Division of Endoscopy Shizuoka Cancer Center Shizuoka Japan; ^3^ Tokyo Metropolitan Institute of Medical Science Tokyo Japan; ^4^ Division of Pathology Shizuoka Cancer Center Shizuoka Japan; ^5^ Shizuoka Cancer Center Research Institute Shizuoka Japan; ^6^ SRL Inc. Tokyo Japan; ^7^ Department of Genetic Counseling Graduate School of Ochanomizu University Tokyo Japan

**Keywords:** exome sequencing, Lynch syndrome, mismatch repair gene, next generation sequencing, pathogenicity, variant of uncertain significance

## Abstract

**Background:**

Lynch syndrome (LS) is the commonest inherited cancer syndrome caused by pathogenic variants of germline DNA mismatch repair (g.*MMR*) genes. Genome‐wide sequencing is now increasingly applied for tumor characterization, but not for g*.MMR*. The aim of this study was to evaluate the incidence and pathogenicity of g.*MMR* variants in Japanese cancer patients.

**Methods:**

Four g.*MMR* genes (*MLH1*, *MSH2*, *MSH6*, and *PMS2*) were analyzed by next generation sequencing in 1058 cancer patients (614 male, 444 female; mean age 65.6 years) without past diagnosis of LS. The g.*MMR* variant pathogenicity was classified based on the ClinVar 2015 database. Tumor MMR immunohistochemistry, microsatellite instability (MSI), and *BRAF* sequencing were also investigated in specific cases.

**Results:**

Overall, 46 g.*MMR* variants were detected in 167 (15.8%) patients, 17 likely benign variants in 119 patients, 24 variants of uncertain significance (VUSs) in 68 patients, two likely pathogenic variants in two patients, and three pathogenic variants in three (0.3%) patients. The three pathogenic variants included two colorectal cancers with MLH1 loss and high MSI and one endometrial cancer with MSH6 loss and microsatellite stability. Two likely pathogenic variants were shifted to VUSs by ClinVar (2018). One colon cancer with a likely benign variant demonstrated MLH1 loss and *BRAF* mutation, but other nonpathogenic variants showed sustained MMR and microsatellite stability.

**Conclusions:**

Universal sequencing of g.*MMR* genes demonstrated sundry benign variants, but only a small proportion of cancer patients had pathogenic variants. Pathogenicity evaluation using the ClinVar database agreed with MSI, MMR immunohistochemistry, and *BRAF* sequencing.

## Graphical abstract

This is the first Japanese paper analyzing more than 1000 cancer patients on germline mismatch repair (MMR) genes and associated molecular assays followed by counseling. Pathogenicity evaluation of MMR genes judged by ClinVar 2018 but not by that of 2015 was compatible with the MMR immunohistochemistry, microsatellite instability status, and *BRAF* mutation.

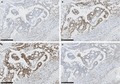

**Abbreviations**
ACMG: American College of Medical Genetics
CI: conflicting interpretation
CRC: colorectal cancer
EC: endometrial cancer
EGAPP: evaluation of genomic application in practice and prevention
ExAC: Exome Aggregation Consortium
g.*MMR*: germline mismatch repair gene
HGMD: Human Gene Mutation Database
HGVD: Human Genetic Variation Database
IHC: immunohistochemistry
LS: Lynch syndrome
MMR: DNA mismatch repair
MSI: microsatellite instability
MSI‐H: high frequency of microsatellite instability
MSI‐L: low frequency of microsatellite instability
MSS: microsatellite stable
NGS: next generation sequence
PolyPhen‐2: Polymorphism Phenotyping V2
Project HOPE: project of high‐tech omics‐based patient evaluation
ToMMo: The Tohoku Medical Megabank Organization
VUS: variant uncertain for significance
WES: whole exome sequencing

## 1 INTRODUCTION

Lynch syndrome (LS; OMIM 120435) is an autosomal dominant cancer predisposition syndrome caused by germline variants in DNA mismatch repair (*MMR*) genes (eg, *MLH1*, *MSH2*, *MSH6*, and *PMS2*). It accounts for 1%‐4% of colorectal cancer (CRC)^1^, ^2^, ^3^, ^4^ and for 2%‐6% of endometrial cancers (EC).^5^, ^6^, ^7^ Variant carriers are at risk of early onset CRC,^3^, ^4^ EC,^5^, ^6^ upper tract urothelial cancers,^8^, ^9^ gastric cancer (particularly in Asian countries, such as Japan and Korea^10^) and a spectrum of other tumors.^11^, ^12^

The classical diagnosis of LS first lists high‐risk individuals by their own cancer history and family cancer history, based on the Amsterdam II criteria^1^ or revised Bethesda guidelines.^2^ These patients are further evaluated by microsatellite instability (MSI) analysis and immunohistochemistry (IHC) of MMR proteins in their tumors, and a final diagnosis of the deleterious variant of the germline *MMR* (g.*MMR*) genes is made by DNA sequencing.^1^, ^2^ Currently, the strategy for LS detection has been shifted toward universal screening using MMR IHC and/or MSI analysis for all or for age‐limited conditions of LS‐related cancers, as this strategy is more sensitive than selection based only on demographic and clinical information.^6^, ^7^, ^13^, ^14^, ^15^ At present, genetic examination of g.*MMR* genes, which is essential for the diagnosis of LS, has been applied to highly suspect candidates with LS‐related cancers, but not to all cancer patients.

Today, oncological characterizations^16^ and diagnosis of inherited cancer syndromes^17^, ^18^ are increasingly obtained by genome‐wide or gene‐panel DNA sequencing using next generation sequencing (NGS).^19^, ^20^ However, these analyses may further complicate the issues regarding the interpretation of variants of uncertain significance (VUSs).^21^ Pathogenicity evaluations of g.*MMR* genes variants at the experimental level have included splicing assays, high‐performance liquid chromatography assays of denatured material, and several other functional assays at the experimental level, in addition to segregation assays and in silico assays.^21^, ^22^, ^23^ Results of these analyses and literature data have been gathered and integrated in large databases, such as ClinGen,^17^, ^18^ which are now easily accessed by clinicians and counselors through ClinVar (https://www.ncbi.nlm.nih.gov/clinvar/).

To date, only a few papers have reported the universal sequencing of g.*MMR* genes using NGS in a large number and variety of cancer patients. The present study has focused on the incidence and pathogenicity evaluations of g.*MMR* variants generated by universal sequencing in Japanese cancer patients who had not been previously diagnosed with LS.

## 2 MATERIALS AND METHODS

### 2.1 Study design and evaluation of *mismatch repair gene* variants

In January 2014, the Shizuoka Cancer Center started a project of high‐tech omics‐based patient evaluation (Project HOPE) that performs genome‐wide exome sequencing in germline and somatic DNAs of various cancer patients.^24^ The current study, as part of the HOPE project, analyzed 1058 cancer patients (614 male and 444 female, mean age ± SD: 65.6 ± 12.9 years old, median: 67 years old, range: 11‐90 years old), who underwent surgical resection at Shizuoka Cancer Center Hospital during January 2014 and March 2015. Cancer cases included 355 colorectal, 129 gastric, 29 pancreatic, 14 brain, 13 ovarian, 10 endometrial, 8 small intestine (including 5 duodenal), and 4 biliary cancers (Table 1); all cancers were pathologically confirmed from surgical samples. At the initial hospital visit, patients and their families filled out the questionnaire sheets on the information of patient's past disease history, family history, and lifestyle aspects. The nurses reconfirmed the content of sheets by conducting interviews for about 20‐30 minutes with each patient.

**Table 1:** Cancer types of 1058 cancer cases

Cancer type	n
Colon	355
Lung	179
Stmach	129
Head and neck	91
Breast	80
Liver	62
Pancreas	29
Kidney	15
Brain	14
Ovary	13
Soft tissue	12
Esophagus	12
Uterus body	10
Uterus cervix	9
Skin	9
Small intestine^a^	8
Biliary tract	4
Others	27

a Cancers of small intestine includes five duodenal cancers.

**Figure 1:**
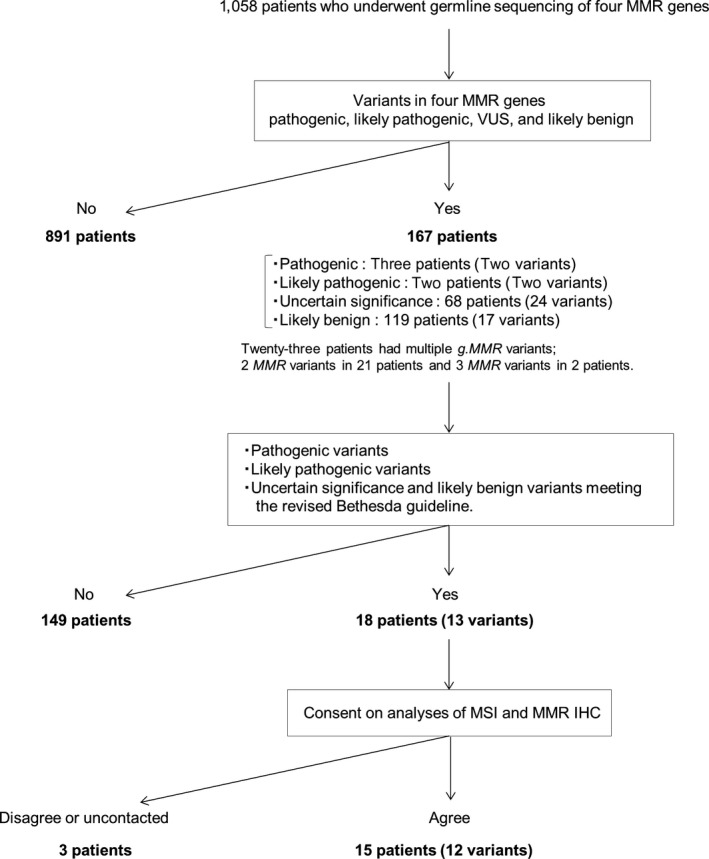
Flowchart for selection for microsatellite instability analysis and DNA mismatch repair (MMR) immunohistochemistry in 1058 cancer cases who underwent germline *MMR* sequencing

Germline DNA, extracted from blood samples obtained soon before the surgery, was subjected to whole exome sequencing (WES). We examined whole exon as well as short length of adjacent noncoding intervening sequence (intron). When a variant was detected, its pathogenicity was determined by referring to the public genome database, ClinVar, reported in July 2015. The pathogenicity was divided into the following five levels: benign, likely benign, VUS, likely pathogenic, and pathogenic.^21^ In addition to known pathogenic variants, unreported genetic variants expected to cause the disorder—the so‐called “expected pathogenic” variants^25^ in the American College of Medical Genetics (ACMG) classification—were also treated as pathogenic.^21^ In silico data, based on Human Gene Mutation Database (HGMD), PolyPhen‐2, SIFT, and allele frequencies reported by ExAC,^21^ the Tohoku Medical Megabank Organization (ToMMo),^26^ and the Human Genetic Variation Database (HGVD)^27^ in 2018, were listed for reference (Table S1). The incidence of cases meeting the revised Bethesda guideline^28^ was compared among three pathogenic levels (1: pathogenic and likely pathogenic, 2: VUS, 3: likely benign, benign, and nonvariants). When pathogenic and likely pathogenic variants were confirmed in g*.MMR* genes, a medical geneticist (H.M) and/or genetics counselors (Y.K, Y.H, and S.H) disclosed the results to the participants^29^ and examined for MSI and MMR protein immunohistochemistry (MMR IHC) (Figure 1). In VUSs, if the patient was contactable and consented after the turnaround of their g*.MMR* data, at least one case with each VUS who met the revised Bethesda guideline was also examined for MSI and MMR IHC. When the pathogenicity of the variant was suspected, a medical check or surveillance of LS was provided as needed.

The Institutional Review Board of Shizuoka Cancer Center ethically approved this study and all the procedures were conducted in accordance with the Helsinki Declaration. Written informed consent of this study was obtained from all participants before study entry and before further LS diagnostic procedures.

### 2.2 Germline DNA analysis

Germline DNA was extracted from blood samples obtained soon before the surgery using a QIAamp DNA Blood Kit (QIAGEN, Venlo, Netherlands). WES was performed using an Ion Torrent AmpliSeq Exome RDY Panel kit (Thermo Fisher Scientific, Waltham, MA, USA), following the manufacturer's recommended protocol.^30^, ^31^ Briefly, 10 ng of DNA was used to prepare the template, and the libraries were prepared automatically using the Ion Chef System (Thermo Fisher Scientific). Libraries were quantified using the quantitative polymerase chain reaction, and 7 pM was sequenced using the Ion Torrent Proton Sequencer (Thermo Fisher Scientific) semiconductor DNA sequencer according to the manufacturer's protocol. The threshold of WES was set to 30%, and <30% of allele ratio was not judged to be germline variants. Germline DNA was first analyzed by WES, and the possibly deleterious variants (pathogenic variants, likely pathogenic variants, and VUSs) were all confirmed by Sanger sequencing.

### 2.3 Microsatellite instability analysis

MSI was analyzed after acquisition of a separate written informed consent for diagnosis of LS. MSI analysis was entrusted to FALCO HOLDINGS Co., Ltd. (Kyoto, Japan) and performed using MSI Analysis System, Version 1.2 (Cat. # MD1641, Promega Corporation, Madison, WI, USA) following the manufacturer's recommended protocol. Briefly, DNA was extracted from FFPE tissue slide by QIAamp DNA FFPE Tissue Kit (QIAGEN). Twenty‐nanograms of DNA was used in a total of 10 µL PCR reaction mix and PCR was performed by Veriti thermal cycler by following cycling profile: 1 cycle 95°C for 11 minutes; 1 cycle 96°C for 1 minute; 10 cycles ramp 100% to 94°C for 30 seconds, ramp 29% to 58°C for 30 seconds, ramp 23% to 70°C for 1 minute; 20 cycles ramp 100% to 90°C for 30 seconds, ramp 29% to 58°C for 30 seconds, ramp 23% to 70°C for 1 minute; 60°C for 30 minutes final extension; and 4°C hold. PCR amplicon was diluted by distilled water and applied to 3130xl Genetic Analyzer (Thermo Fisher Scientific). Fragment analysis was performed by GeneMapper software (Thermo Fisher Scientific) and MSI status was evaluated by comparing normal and tumor tissue using five nearly monomorphic mononucleotide microsatellite markers (BAT25, BAT26, NR21, NR24, and MONO27). A high frequency of microsatellite instability (MSI‐H) was defined when the tumor DNA demonstrated instability in two or more markers, whereas a low frequency of MSI instability (MSI‐L) was defined when only one marker was instable, and microsatellite stability was defined when a null marker showed instability.

### 2.4 Immunohistochemistry of mismatch repair protein

Paraffin‐embedded block of the surgical specimen was sliced into 3 μm thickness and was attached onto a slide glass. After deparaffinization with xylene for 15 minutes and stepwise de‐xylene treatment by ethanol, heat treatment was performed using Epitope Retrieval Solution 2 (pH 9.0, Leica Biosystems, Wetzlar, Germany) at 95°C for 20 minutes to activate antigenicity. As a primary antibody against each MMR protein, anti‐hMLH1 antibody (Clone ES05, ×50 dilution, Dako, Santa Clara, CA, USA), anti‐hMSH2 antibody (Clone FE11, ×50 dilution, Dako), anti‐hMSH6 antibody (Clone EP49, ×50 dilution, Dako), anti‐hPMS2 antibody (Clone EP51, ×25 dilution, Dako) were used. The secondary antibody was reacted at room temperature for 8 minutes using Bond Polymer Refine Detection (Cat. No. DS9800, Leica). Color was developed with diaminobenzidine (SIGMA, St. Louis, MO, USA) for 10 minutes at room temperature. When MMR protein was not expressed or its expression was severely repressed in the cancer tissue, contrasting with the diffuse expression in the noncancer tissue, the tumor was designated as MMR expression negative. This evaluation was done by the expert pathologist (T.O). Histological images were taken using a pathological slide scanner (NanoZoomer S360 Digital slide scanner, C 9600‐02, Hamamatsu Photonics KK, Shizuoka, Japan).

### 2.5 Statistical analysis

Incidences of CRC patients meeting the revised Bethesda guideline were compared among the three pathogenic levels using Spearman rank correlation test and the JMP ver.12.2.0 statistical software. *P* < .05 was considered to be statistically significant.

## 3 RESULTS

### 3.1 Incidence of germline *mismatch repair gene* variants

Based on the ClinVar database from 2015, the current WES of g.*MMR* genes in 1058 cancer patients demonstrated three (0.3%) pathogenic variants (*MLH1* c.545 + 2T>C, *MLH1* c.2041G>A [p.Ala681Thr], and *MSH6* c.1126G>T [p.Glu376*]) in three patients, two (0.2%) likely pathogenic variants (*MLH1* c.453G>A [p.Thr151=] and *MLH1* c.1153C>T [p.Arg385Cys]) in two patients (Table 2), 24 VUSs in 68 patients, and 17 likely benign variants in 119 patients (Table S1). Of three pathogenic variants, the pathogenicity level and allele frequency of two variants (*MLH1* c.545 + 2T>C^32^ and *MSH6* c.1126G>T) were not registered in the ClinVar, HGMD, or ExAC databases. However, they were splice sites variants and nonsense variants, and had very strong evidence of pathogenicity (PVS1 category).^21^ The pathogenicity level evaluated by ClinVar, either in 2015 or in 2018, was not consistent with the HGMD category or other in silico assays (Polyphen‐2 and SIFT). In total, 23 patients had multiple g*.MMR* variants: two variants in 21 patients and three variants in two patients. Variants with allele frequencies ≥0.5%, which are generally judged as nonpathogenic, were reported by ExAC in none of 34 of the nonpathogenic variants (VUSs + likely benign variants) and reported by HGVD in five of 23 (21.7%) nonpathogenic variants. The Japanese database (ToMMo) still lacks information for currently recognized g.*MMR* variants in the healthy populations (Table S1).

**Table 2:** Demographics, microsatellite instability, and mismatch repair protein expression in patients with germline mismatch repair genes variants

								IHC of MMR protein					
Pathogenicity (2015)^a^	No.	Variants	Gender	Age (y.o)	Tumor site	Revised Bethesada criteria	MSI	MLH1	MSH2	MSH6	PMS2	Somatic *BRAF* V600E	Pathogenicity (2018)^a^
Pathogenic	1	*MLH1* (c.545 + 2T>C)	M	29	Rectum	Yes	MSI‐H	(−)	(+)	(+)	(−)	−	NR
	2	*MLH1* (c.2041G>A)	F	52	Cecum	Yes	MSI‐H	(±)	(+)	(+)	(±)	−	Pathogenic
	3	*MSH6* (c.1126G>T)	F	55	Endometrium	No	MSS	(+)	(+)	(−)	(+)	−	NR
LP	4	*MLH1* (c.453G>A)	M	44	Lung	No	MSS	(+)	(+)	(+)	(+)	−	US
	5	*MLH1* (c.1153C>T)	F	73	Sigmoid colon	No	MSS	(+)	(+)	(+)	(+)	−	US
US	6	*MLH1* (c.46G>C)	M	67	Sigmoid colon	Yes	MSS	(+)	(+)	(+)	(+)	−	US
	7	*MSH6* (c.3772C>G)	M	59	Transvers colon	Yes	MSS	(+)	(+)	(+)	(+)	−	US
LB	8	*MLH1* (c.1990‐6G>A)	M	46	Rectum	Yes	Uncontacted^b^	Uncontacted				−	CI (US1 LB3)
	9		M	51	Rectum	Yes	MSS	(+)	(+)	(+)	(+)	−	
	10		M	51	Appendix	Yes	Uncontacted	Uncontacted				−	
	11		F	38	Sigmoid colon	Yes	MSS	(+)	(+)	(+)	(+)	−	
	12	*MSH2* (c.471C>A)	F	51	Sigmoid colon	Yes	MSS	(+)	(+)	(+)	(+)	−	LB
	13	*MSH2* (c.972G>A)	F	78	Transvers colon	Yes	Disagree^c^	Disagree				−	LB
	14	*MSH2* (c.1255C>A)	F	40	Rectum	Yes	MSS	(+)	(+)	(+)	(+)	−	LB
	15		M	53	Rectum	Yes	MSS	(+)	(+)	(+)	(+)	−	
	16	*MSH6* (c.532C>T)	F	51	Ascending colon	Yes	MSS	(+)	(+)	(+)	(+)	−	CI (US3 LB1)
	17		F	61	Ascending colon	Yes	MSI‐H	(−)	(+)	(+)	(−)	+	
	18	*MSH6* (c.3246G>A)	M	79	Rectum	Yes	MSS	(+)	(+)	(+)	(+)	−	CI (US2 LB3 B1)

Abbreviations: CI, conflicting interpretation of pathogenicity; F, female; IHC, immunohistochemistry; LB, likely benign; LP, likely pathogenic; M, male; MMR, DNA mismatch repair; MSI‐H, high frequency of microsatellite instability; MSS, microsatellite stable; NR, not reported; US, uncertain for significance.

a Pathogenicity level was determined using ClinVar database at 2015 and ACMG guideline, and ClinVar database at 2018 alone.

b Patient have uncontacted hospital for genetic counselling.

c Patient disagreed on MSI examination.

Of 355 colorectal cancer cases, 71 cases met the revised Bethesda guideline. Colorectal cancer cases meeting the revised Bethesda guideline were recognized in two (66.7%) of the three cases with pathogenic and likely pathogenic variants, two (7.4%) of the 27 cases with VUSs, and 67 (20.6%) of the 325 cases with benign genotypes (including likely benign and benign variants and nonvariants; *P* = .375).

### 3.2 Microsatellite instability and mismatch repair protein expression in cases with various germline mismatch repair variants

MSI analysis and MMR protein IHC were performed for the cases that met the conditions described in Figure 1 and that provided consent for these analyses. In total, 15 patients (12 variants) agreed to undergo MSI analysis and MMR IHC, but three patients did not agree or did not contact our hospital after the turnaround of variant data (cases 8, 10, and 13; Table 2).

**Figure 2:**
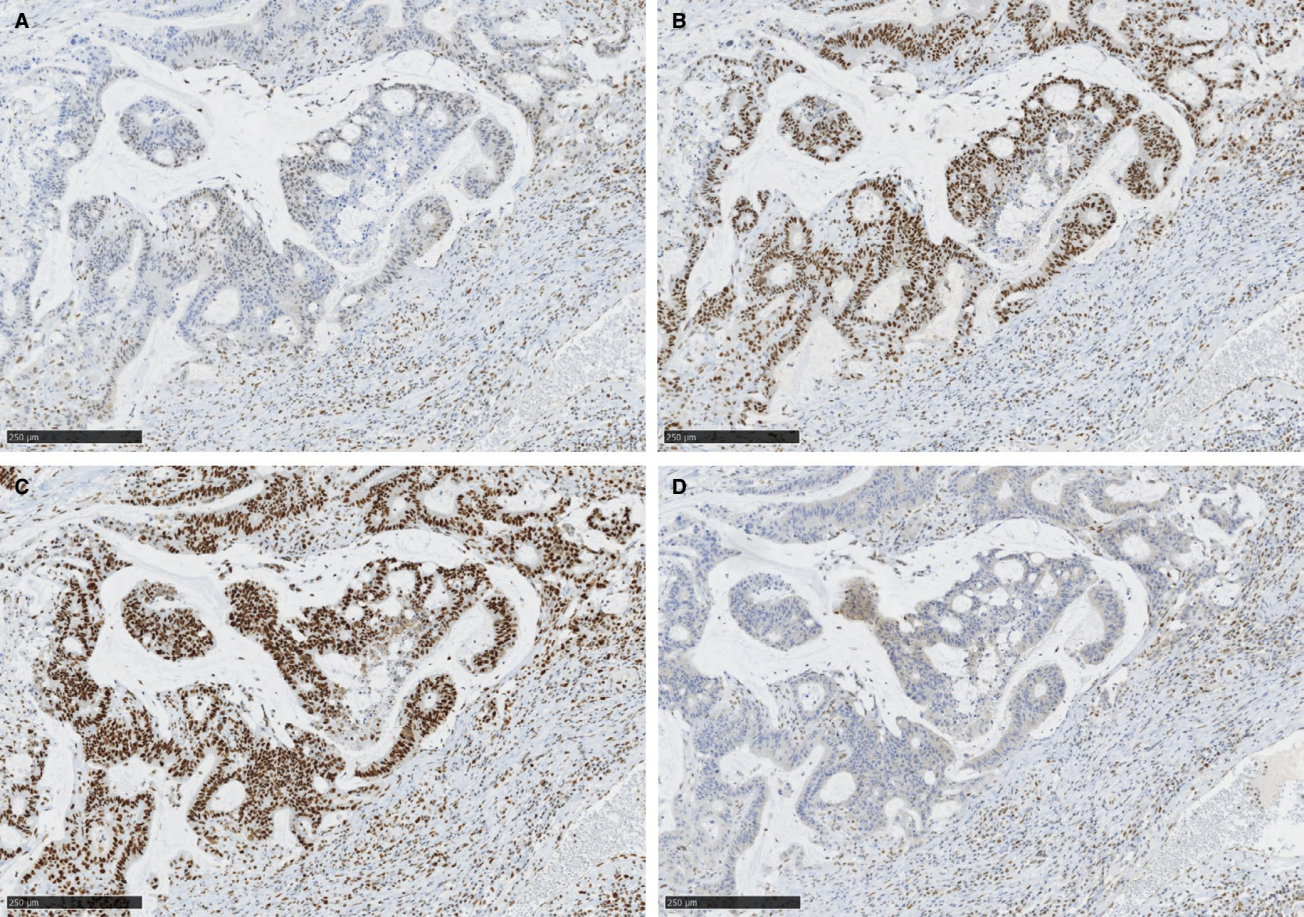
Mismatch repair protein expression in the invasive mucinous carcinoma of case 2, with the germline pathogenic variant of *MLH1* showing severely repressed expression of MLH1 and PMS2 (×50, A: MLH1, B: MSH2, C: MSH6, and D: PMS2, scale bars indicating 250 μm)

A subject with cecal cancer (case 2) who showed histological signs of partially mucinous differentiation had a pathogenic variant of g.*MLH1* c.2041G>A, a high frequency of MSI (5 of 5 markers), repressed MLH1 and PMS2 expression (Figure 2). A subject of endometrial cancer (case 3) with a germline pathogenic variant (*MSH6* c.1126G>T) showed a loss of MSH6 expression, but retained microsatellite stability, suggestive of an *MSH6*‐specific phenomenon.^33^ Case 17, with a likely benign variant (*MSH6* c.532C>T), revealed preserved tumor MSH6 and MSH2 expression, but showed MSI‐H and a loss of tumor expression of MLH1 and PMS2. In this tumor, a *BRAF* V600E mutation was recognized. Overall, the IHC and MSI statuses analyzed in 12 g.*MMR* variants (15 patients) were all compatible with the ClinVar database in 2018 (Table 2).

### 3.3 Changes in variant categories evaluated by the ClinVar 2015 and 2018 databases

Two cases judged as likely pathogenic variants based on ClinVar database edited in 2015 (case 4: lung cancer case with *MLH1* [c.453G>A]; and case 5: sigmoid colon cancer case with *MLH1* [c.1153C>T]) were re‐categorized as VUSs by the ClinVar 2018 database. These cases contained four MMR proteins and showed microsatellite stability (Table 2). Similarly, five previously designated VUSs were re‐categorized as “conflicting interpretation (CI)” variants in 2018, and nine likely benign variants were shifted to three VUSs, five CIs, and one benign/likely benign variant (Table 3 and Table S1).

**Table 3:** Change of pathogenicity evaluation of germline mismatch repair gene variants from 2015 to 2018

Pathogenicity level based on the ClinVar database		
2015	2018	n
Likely pathogenic	VUS	2
VUS	CI	5
Likely benign	VUS	3
Likely benign	CI	5
Likely benign	Benign/Likely benign	1

Abbreviations: CI, conflicting interpretations of pathogenicity; VUS, variant uncertain for significance.

## 4 DISCUSSION

The current study describes universal germline *MMR* (g.*MMR*) exome sequencing on 1058 cancer cases, and demonstrated pathogenic variants in 0.3% (3) of all cancer cases, 0.6% (2/355) of the colorectal cancer cases, and 10% (1/10) of the EC cases. The worldwide incidences of LS have been reported as 1.0%‐3.7% in CRC patients and 1.7%‐5.9% in EC patients. Subtle differences are seen among various countries for both CRCs (1.0%^34^ to 1.9%^35^ in the USA, 0.7%^4^ to 3.1%^14^ in Spain, 2.4%^13^ to 3.7%^36^ in France, 0.6%^37^ in Australia, and 0.7%^38^ in Japan) and in EC (1.7%^7^ to 4.5%^39^ in the USA, 5.9%^6^ in Canada, 4.6%^40^ in Spain, 2.4%^5^ in Australia, and 2.9%^41^ in Japan). The incidence of g.*MMR* variants in Japanese CRC patients tends to be lower compared with other countries. A variety of founder mutations of *MMR* genes have been reported over the world. A nationwide study conducted by the Japanese Society for Cancer of the Colon and Rectum reported that large deletions or duplications were common (26.6%) in Japanese LS patients including the *MLH1* founder mutations.^42^ Therefore, when only sequencing analysis is performed, these variants may be missed. Families with Japanese founder mutations may be limited to biased areas of the country, and the true prevalence of Japanese LS should be investigated in nation‐wide studies. In addition, a lifetime risk of developing CRC among carriers of the mutations may be influenced by environmental factors and the lifestyles.^43^

Within our literature survey, this is the first study analyzing g.*MMR* variants of more than 1000 cancer patients in Japan. The Tohoku Medical Megabank Organization (ToMMo) determined allele frequencies of g.*MMR* variants in healthy Japanese cohorts and reported 0.6% pathogenic variants (13/2049; *MLH1*: 0.49%, *MSH2*: 0.08%, *MSH6*: 0.05%, and *PMS2*: 0%).^26^ This incidence seems to be compatible with the current result (0.3%), as the risk of developing any cancer is high in g.*MMR* variant carriers (at age 70, male: 75%, female: 58%).^44^ The NGS data from the USA demonstrated similar incidences of pathogenic g.*MMR* variants in advanced cancer patients (0.7% [11/1566] at the Memorial Sloan Kettering Cancer Center^19^ and 0.5% [5/1000] at MD Anderson Cancer Center^20^).

Until the early 2000s, screening of LS was done by focusing on cancer patients with high‐risk conditions according to the Amsterdam II criteria or revised Bethesda guideline;^28^ and MMR IHC and MSI analysis were then conduced on these selected patients.^2^, ^34^, ^36^ Later, universal screening using MMR IHC^7^, ^45^ and/or MSI analysis^36^, ^46^ (±age limitation) prevailed over these demographic selections. The earlier screening strategy desensitized the LS detection ratio when compared to universal screening (eg, the revised Bethesda guideline gave a rate of 0%‐50%^5^, ^7^, ^47^ and the Society of Gynecologic Oncology criteria gave a rate of 43%^15^ in the setting of 100% sensitivity by universal screening). These data were compatible with the current result of no difference in the ratio of colorectal cancer cases meeting the revised Bethesda guideline among the three variant levels. Although the cost‐effectiveness is controversial,^34^, ^48^ the Evaluation of Genomic Application in Practice and Prevention (EGAPP) working group in the USA does not currently recommend the use of family history to exclude individuals with newly diagnosed cancers from offers of genetic testing, because of the poor identification of LS. Thus, the trend to detect LS has shifted toward the use of universal screening.^49^

When compared with MMR IHC^34^, ^37^, ^50^ and MSI analysis,^13^, ^36^ the sensitivity of detecting LS is equivalent to MSI analysis when using four MMR proteins for IHC.^51^ Approximately 3% of the cases were discordant in these two methods, and 5% of cancers that demonstrated MSI had normal MMR protein expression,^52^, ^53^ while some tumors with pathogenic variants of g.*MSH6* revealed microsatellite stability,^33^ like case 3 in the current study (Table 2). IHC is readily available and generally inexpensive and can predict the causative gene; therefore, it is presently considered the optimal first‐line screening tool rather than the MSI test.^51^ In tumors with repressed expression of MLH1 and PMS2, further analysis of *BRAF* mutation or *MLH1* promoter methylation is needed to exclude somatic *MLH1* alterations,^54^ as in case 17 (Table 2).

Today, cancer genomic‐based precision medicine has led to the use of NGS for universal somatic^55^ and germline^19^, ^20^ *MMR* sequencing for selected‐gene or genome‐wide analysis. This strategy saves the initial step of IHC screening and directly examines the genome; therefore, it may reduce costs if the charge for the genetic test decreases. A study by Gould‐Suarez et al comparing the cost‐effectiveness of 10 strategies for detecting LS demonstrated that parallel testing with MSI and MMR IHC offered the most robust yield at a reasonable cost, and that universal g.*MMR* sequencing was the most expensive. However, they concluded that germline testing could be the most cost‐effective test if the price of g.*MMR* sequencing were to decrease to $633–$1518,^56^ which could be realized given the recent trends in NGS.^57^

However, the main issue of universal sequencing of g.*MMR* involves the pathogenicity evaluation based on the public genome database, due to the high proportion of VUSs and the transition to a pathogenic level with time. In fact, the current study using the ClinVar database demonstrated 24 types of VUSs in 68 patients (Table S1), and 16 variants changed their variant levels between 2015 and 2018 (Table 3). Notably, two variants judged as probably pathogenic in 2015 (case 4 and case 5 in Table 2) were shifted to VUSs in 2018. Moreover, not all the pathogenic variants have been clarified in the database, and entirely new pathogenic variants are still being discovered (case 1^32^ and case 3 in Table 2). Cases of null variants (such as nonsense, frameshift, and splice site mutations) can be easily judged as pathogenic, but difficulties arise in cases with moderate to strong levels of pathogenicity showing novel missense variants and the same amino acid‐type variants, and these need functional assay validation.^21^ Conversely, MMR IHC and MSI analysis can be helpful for g.*MMR* variants with undetermined pathogenicity, as the results are highly concordant with the ClinVar‐based pathogenicity level, according to the current study findings. Accumulation of these biomedical data is essential for accurate and reliable genetic evaluation in the future.

The current study has some limitations. It was conducted at a single cancer center in Japan. More than 1000 cases, but not a particularly large number of subjects, were included in order to detect rare events. In addition we used the Ion Torrent system for NGS, which gives a higher throughput (80‐100 Mb/hour) in the 100‐bp mode but tends to produce homopolymer‐associated indel errors when compared with the MiSeq system (Illumina, San Diego, USA).^58^ Since we have analyzed only whole exon and adjacent short sequences of introns, we cannot observe all pathogenic variants of intron sites. In addition, we did not exclude a possibility of false positive/negative variant of *PMS2* due to the presence of highly homologous pseudogenes in our method.

In conclusion, universal sequencing of g.*MMR* genes demonstrated a number of benign variants, as well as definitive pathogenic variants in a small fraction of cancer patients. Pathogenicity evaluation using the ClinVar database was highly concordant with MSI analysis, MMR immunohistochemistry, and *BRAF* sequencing. Reliable pathogenicity evaluation of these variants requires further accumulation of biomedical information for each variant.

## CONFLICT OF INTEREST

The authors declare that they have no conflict of interest.

## AUTHOR CONTRIBUTIONS

YK, YH, SH, and HM worked on genetic counseling and patient management; TO and MA performed pathological sample preparation and diagnosis; SO, KU, and MK worked on DNA sequencing and laboratory tasks; TN was in charge of bioinformatics work in genetic evaluation; YK and HM wrote the manuscript; and KY supervised the study.

## Data Availability

The g.*MMR* variant data of this study are available in the National Bioscience Database Center (NBDC) and Japanese Genotype‐phenotype Archive (JGA) databases under the accession number hum0186 and JGAS00000000183, respectively.
